# *Beauveria bassiana* associated with a novel biomimetic hydrogel to control *Aedes albopictus* through lure and kill ovitraps

**DOI:** 10.1002/ps.8476

**Published:** 2024-10-17

**Authors:** Marco Friuli, Riccardo Paolo Lia, Paola Nitti, Leonardo Lamanna, Domenico Otranto, Marco Pombi, Christian Demitri, Claudia Cafarchia

**Affiliations:** ^1^ Department of Engineering for Innovation University of Salento Lecce Italy; ^2^ Department of Veterinary Medicine University of Bari Bari Italy; ^3^ Department of Veterinary Clinical Sciences City University of Hong Kong Hong Kong Hong Kong; ^4^ Dipartimento di Sanità Pubblica e Malattie Infettive Università di Roma “Sapienza” Rome Italy

**Keywords:** hydrogels, biocompatibility, biomimetics, bioinsecticides, *Aedes albopictus*, *Beauveria bassiana*, mosquitoes

## Abstract

**BACKGROUND:**

Within the framework of sustainable and effective control methods for *Aedes albopictus*, two different conidial suspensions, *Bb*CS‐1 and *Bb*CS‐2 (respectively without and with nutrients), were used as solvents for the biopolymers water‐soluble 2‐hydroxyethylcellulose (HEC) and sodium alginate (SA). In this way, two different classes of hydrogels were prepared for each polymer (previously shown to attract tiger mosquito oviposition) to produce HEC‐based and SA‐based *Bb*/Gel systems with and without nutrients. The aim was to achieve a long‐lasting and cost‐effective lure‐and‐kill oviposition substrate useful for lethal ovitraps. *Beauveria bassiana* (*Bb*) survival and growth in the different *Bb*/Gel systems were monitored for 24 days. Following the growth assay, 24‐day‐old *Bb*/Gel systems were tested against *Ae. albopictus* eggs through a hatching test to evaluate their lethal effect.

**RESULTS:**

Gel systems enhance *Bb*'s longevity (up to 24 days) more effectively than standard liquid conidial suspensions, proving that tested HEC‐ and SA‐based hydrogels are not toxic for *Bb* (biocompatibility) and create a microenvironment suitable to sustain prolonged fungal growth. In particular, the results indicate that gel system based on hydroxyethylcellulose is a suitable delivery substrate for supporting the activity of *Bb* and is simultaneously effective against *Ae. albopictus* eggs through a combined mechanism of mechanical effect and fungal action (CM > 90%).

**CONCLUSION:**

The efficacy of *Bb* gel systems was assessed according to its properties in favouring the growth and vitality of *Bb* as well as in reducing the *Ae. albopictus* hatching eggs rate. Further studies, in semi‐field and field conditions, will be useful to evaluate the efficacy of *Bb*/Gel systems on adults in terms of attraction, oviposition, mortality, and potential autodissemination to propose a new tool in precision pest management. © 2024 The Author(s). *Pest Management Science* published by John Wiley & Sons Ltd on behalf of Society of Chemical Industry.

## Graphical abstract

This study introduces a biomimetic hydrogel with *Beauveria bassiana*, sustaining fungal growth over 24 days. The resulting *Bb*/Gel system effectively combines mechanical trapping with bioinsecticide action, registering over 90% mortality in *Aedes albopictus* eggs and larvae.

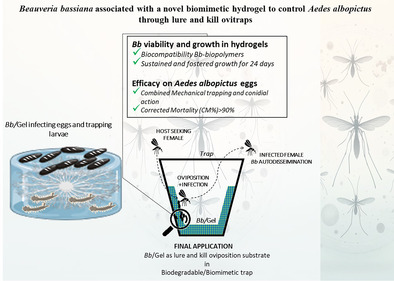

**ABBREVIATIONS**
*Bb*
: *Beauveria bassiana*
CS: conidial suspension
HEC: 2‐hydroxyethilcellulose
LOT: lethal ovitrap
Pda: potato dextrose agar
SA: sodium alginate

## 1 INTRODUCTION

Despite efforts to develop alternative vector control strategies, chemical insecticides remain the primary tool to control vector‐borne diseases (e.g., ground or areal application by spraying).^1^ This overreliance on chemical interventions has triggered the emergence and spread of insecticide resistance across various arthropod vectors, posing a growing threat to human, animal, and environmental health, as well as important economic commitment for their control.^2^, ^3^ In response to the environmental and health risks, more restrictive pesticide regulations^4^, ^5^ have led to investigation of alternative, low‐impact, and cost‐effective methods and strategies for the control of arthropod vectors.^6^, ^7^, ^8^, ^9^, ^10^ Among major threats to human health, *Aedes albopictus* has been recognized as a global vector of several arboviruses (i.e., Chikungunya virus, Zika virus, Dengue virus and yellow fever virus) due to its spreading to temperate and tropical areas in all continents.^11^, ^12^, ^13^, ^14^, ^15^, ^16^ The intervention strategies against this mosquito vector are mainly based on the use of insecticides and insect growth regulators, despite the growing evidence of insecticide resistance in this species.^17^ Ovitraps, which have been traditionally employed as a monitoring tool,^18^, ^19^ showed potential as a massive control method when coupled with an insecticide (lethal ovitrap, LOT) with some positive results in large‐scale experimental campaigns.^20^ However, their cost (overall related to monitoring, maintenance, and retrieval after use) are not offset by high performance, which therefore limits their use.^21^ In addition, the trap's performance is strongly influenced by its short duration (i.e., they work properly until substrate is moist) and type of oviposition substrate,^22^ and is reduced by competition with the other sites available in the environment.^23^ New trap variants have been proposed, such as biodegradable construction materials (e.g., starch), oviposition substrates based on paper soaked in an insecticide solution, and synthetic hydrogels (e.g., polyacrylamide hydrogel) combined with active substances to obtain a synergic lasting and killing effect.^22^, ^23^, ^24^, ^25^, ^26^, ^27^ Nevertheless, to overcome the limitations of chemical control, alternative strategies mainly based on biological control agents such as *Bacillus thuringiensis* and entomopathogenic fungi (e.g., *Beauveria bassiana* [*Bb*] and *Metarhizium anisopliae*) have been proposed.^28^, ^29^ Among them, entomopathogenic fungi have been largely investigated due to their safety for human and animal health, and for their autodissemination process when applied in aqueous or oily solutions, granules, pellets, or floating formulations.^30^, ^31^ However, the employment of entomopathogenic fungi for mosquito control is still limited due to their low persistence in field conditions (e.g., exposition to heat, ultraviolet (UV) radiation, and substrate drying).^32^, ^33^ In recent years, innovative strategies have been developed to improve the persistence and vitality of these fungi, such as the encapsulation of fungal conidia in polymeric matrices that can act as a protective barrier against solar irradiation, thermal stress or the addition of hydrophilic/hygroscopic materials to the formulations (e.g., polymers like polyacrylamide gels powders or ceramic materials like zeolites) since they can retain moisture for a longer time, delaying desiccation.^30^, ^31^, ^34^ Furthermore, recent data have demonstrated how polymers in some cases not only preserve their properties but also increase the *Bb*'s conidia production, improving their action.^35^, ^36^, ^37^, ^38^ It was proved that specific hydrogels formulations (without any attractants) can be very effective oviposition substrates for ovitraps when the material is produced with a specific set of parameters able to simulate the characteristics of typical *Ae. albopictus* oviposition sites (the biomimetic approach^39^). For example, macromolecular hydrogel substrates, made of biodegradable and biocompatible polymers (celluloses or alginates), were successfully tested for their oviposition performance with *Ae. albopictus*.^39^, ^40^, ^41^, ^42^, ^43^, ^44^, ^45^, ^46^, ^47^ Since there is an overlapping of feature between what is necessary to attract mosquitoes and what could be useful to protect Bb survival and growth, it is possible to hypothesize transforming a luring substrate into a lure and kill substrate.

In this work we tested hydrogel‐based oviposition substrates combined with a bioinsecticide, choosing *Bb* as a proof of principle. *Bb* was added to a fully biodegradable and biocompatible hydrogel (to obtain a new *Bb*/Gel system) to develop a lure‐and‐kill substrate inside an innovative long‐lasting and chemical‐free LOT for *Ae. albopictus*.

## 2 MATERIALS AND METHODS

### 2.1 Materials

A native strain of *Bb* (CD1123) was obtained from naturally infected ticks collected in a private dog shelter in Putignano (Bari, Italy). The strain was morphologically and molecularly identified as previously described^48^ and maintained on potato dextrose agar (PDA, Liofilchem, Roseto degli Abruzzi (Te) Italy) at 4 °C. Two different natural polymer powders were used for gel preparation: 2‐hydroxyethilcellulose (HEC, average molecular weight 720 000; Aldrich Chemistry, Milano, Italy) and sodium alginate (SA, average molecular weight 120 000; SAFC, Darmstadt, Germania). Pepton (Sigma‐Aldrich, Sigma‐Aldrich, Milano, Italy), Tween 80 (Fluka, Darmstadt, Germania), and chitosan (low molecular weight chitosan flakes; Sigma‐Aldrich, St. Louis, MI, USA) were added to the formulation as nutrients for *Bb*. Potato dextrose agar (PDA; Liofilchem, Roseto degli Abruzzi (Te) Italy) was employed to quantify the growth of *Bb* within the gels and yeast extract (Oxoid, Sigma‐Aldrich, Darmstadt, Germania) was used to improve *Ae. albopictus* hatching rate. All the materials and tools were previously sterilized by UV lamp at 500 W for 45 min under a laminar flow hood or, when possible, autoclaved for 15 min at 121 °C and 3 bar. A Binder climatic chamber was used to incubate samples and was cleaned after each use with an ethanol solution (80% v/v). For hatching assay, 5‐day‐old *Ae. albopictus* eggs from the insectary of the Department of Public Health and Infectious Diseases of Sapienza University of Rome were employed.

### 2.2 Preparation of *Bb* conidia suspension

*Beauveria bassiana* conidia were obtained by culturing 15 *Bb* strains on PDA for 3 weeks at 25 °C. Plates were washed with 10 mL of sterile distilled water containing 0.1% v/v Tween 80 (CS‐1) or with 10 mL of sterile peptone water and containing 1% v/v Tween 80 and 2% in weight (wt%) of not solubilized chitosan flakes (CS‐2) to obtain two different suspensions of *Bb*, *Bb*CS‐1 and *Bb*CS‐2, respectively. The suspensions, which had neutral pH, were vortexed and turbidity was adjusted spectrophotometrically (Biosan DEN 1) to an optical density of 10 McFarland and serially diluted to reach 6–8 McFarland. The amount of conidia for both suspensions was evaluated by quantitative plate counts of Colony Forming Unit per mL (CFU/mL) on PDA plates.

### 2.3 Preparation of *Bb*‐hydrogel systems

*Bb*CS‐1 and *Bb*CS‐2 (previously described) were the solvent for HEC or SA (water‐soluble polymer powders) used to prepare gel systems (HEC‐ or SA‐based *Bb*/Gel) consisting of *Bb* fungal structures and polymer. The systems were prepared at room temperature under sterile conditions by directly adding 16 wt%^39^ of water‐soluble powder (HEC or SA) to *Bb*CS‐1 and *Bb*CS‐2. The mixture was stirred at 1500–2000 rpm until complete solubilization. At the end of preparation the pH was still neutral.

Following this protocol, two classes of *Bb*/Gel systems were obtained for each polymer type (see Table 1). Gels without *Bb*, used in hatching tests as controls as well as in the *Bb* vegetative growth tests, were prepared by following the previously described protocols but adding the polymers to distilled water and CS‐1 or CS‐2, respectively.

**Table 1:** *Bb*/Gel samples and controls prepared and tested

Name of the sample	Composition	Description
CS‐1	H_2_O, Tween 80	Initial suspension without nutrients
CS‐2	Peptone water, Tween 80, chitosan flakes	Initial suspension with nutrients
*Bb* CS‐1	*Bb* + CS‐1	Conidial suspension without nutrients
*Bb* CS‐2	*Bb* + CS‐2	Conidial suspension with nutrients
*Bb* CS‐1 HEC 16	*Bb* + CS‐1 + HEC	HEC‐based *Bb*/Gel system without nutrients
*Bb* CS‐2 HEC 16	*Bb* + CS‐2 + HEC	HEC‐based *Bb*/Gel system with nutrients
*Bb* CS‐1 SA16	*Bb* + CS‐1 + SA	SA‐based *Bb*/Gel system without nutrients
*Bb* CS‐2 SA16	*Bb* + CS‐2 + SA	SA‐based *Bb*/Gel system with nutrients
CS‐2 HEC 16	CS‐1 + HEC	Gel system (used in vegetative growth)
CS‐2 SA16	CS‐2 + SA	Gel system (used in vegetative growth)
HEC16	H_2_O + HEC	Gel control (no *Bb*, no nutrients)
SA16	H_2_O + SA	Gel control (no *Bb*, no nutrients)
Fresh *Bb*	*Bb* + CS‐2	24 h‐old conidial suspension control with nutrients

All the preparations had neutral pH.

### 2.4 *Bb* growth evaluation in the *Bb*/Gel system

The biocompatibility between polymers and *Bb* was assessed by studying the viability and growth of the fungus inside *Bb*/Gels in comparison with *Bb* conidial suspensions (i.e., *Bb*CS‐1 and *Bb*CS‐2). In particular, the bottles containing the *Bb*/Gel samples and conidial suspensions were incubated at 25 ± 1 °C and 90% relative humidity (RH) for 24 days. Growth of *Bb* was assessed every 4 days by evaluating the amount of conidia for every sample through quantitative plate counts of CFU/mL on PDA plates.^34^ The results were expressed as log_10_ of CFU/mL. CS‐1 and CS‐2 solutions were tested as controls.

### 2.5 *Bb* conidial viability assay

*Bb* conidial viability assay was performed on the best performing *Bb*/Gels in growth assay after 24 days of incubation at 25 ± 1 °C and 90% RH. In particular, 0.1 g of gel was harvested from each sample and diluted in 9.9 mL of water. The solution was vortexed, left to settle for 15 min, and the supernatant removed. The remaining suspension was filtered with 8‐μm Watman filters, then centrifuged (3000 *g* × 5 min), washed twice in 1 mL of phosphate‐buffered saline solution (PBS), and re‐suspended in 1 mL of PBS. Finally, the numbers of active conidia were determined by quantitative plate counts of colony forming units (CFU)/mL on PDA after 4 days of incubation at 25 °C and 90% RH. The data were expressed as log_10_ of CFU/mL. All the experiments were performed in duplicate and repeated three times on different days.

### 2.6 *Bb* vegetative growth assay

The vegetative growth of *Bb* on the prepared hydrogels was assessed by placing a *Bb* mycelial plug (i.e., 10 mm in diameter) onto the center of a 90‐mm Petri dish containing gel samples without *Bb* (i.e., CS‐2 HEC16 and CS‐2 SA16) and measuring the diameter of the colonies after incubation at 25 °C for 10 days.^48^ All the experiments were performed in duplicate and repeated three times on different days.

### 2.7 Bioassay on *Ae. albopictus* eggs: hatching test

The lethal effect of *Bb*CS‐1, *Bb*CS‐2 and *Bb*/Gels against *Ae. albopictus* eggs following the growth period was evaluated through a hatching test. The lethal effect of all the 24‐day‐old systems employed in *Bb* growth assay was assessed. Briefly, 2 g of each *Bb*/Gel and 2 mL of each of *Bb*CS‐1 and *Bb*CS‐2 and the respective controls (i.e., CS‐1, CS‐2, *Bb*CS‐1, *Bb*CS‐2, HEC16, SA16, fresh *Bb*CS‐2) were harvested and spread on a sterile paper (20 × 20 × 1 mm, Whatman No. 1, labor 67 g/m^2^; Tecnochimica Moderna) placed inside a 40‐mm Petri dish. Subsequently, 30 *Ae. albopictus* eggs were laid on each prepared paper with the assistance of a sterile feather. Eggs were collected from an *Ae. albopictus* colony raised in an insectary placed at the Parasitology Section of the Department of Public Health and Infectious Diseases of the Sapienza University of Rome. Eggs were collected 24 h before the test. The packets were then incubated for 5, 10, and 15 days (contact periods) at 25 ± 1 °C and 90% RH. At the end of each contact period, the papers were removed from the Petri dishes and transferred to sterile plastic containers filled with 80 mL of tap water and 2 mg of yeast extract to promote hatching. The hatching chamber was incubated in an air‐conditioned room at 24 °C and 80% RH for 5 days, during which time the emerging larvae were counted.^49^ Larvae were monitored for an additional 5 days after the initial count, and in the case of late hatching, new larvae were added to the total count. CS‐1, CS‐2, and 24 h *Bb*CS‐2 (fresh *Bb*) were used as controls. Mortality data were averaged, and the results were expressed as corrected mortality rates (CM%, benchmarked against water) using SchneiderOrelli's formula [e.g., corrected mortality% [CM%] = (mortality% in *Bb*/Gel − mortality% in distilled water control)/(100 − mortality% in distilled water control) × 100], where mortality% = (1 − living larvae/tested eggs) × 100.^48^ All experiments were conducted in duplicate and repeated three times on different days.

### 2.8 Morphological analysis of eggs

The main purpose of the analysis was to evaluate the eggs' morphological conditions, the presence of fungal structures on them, and the eventual material‐related effects on eggs and larvae. Eggs from different *Bb*/Gel systems after 5, 10, and 15 days of contact at 25 ± 1 °C and 90% RH were observed with an optical microscope (Axio Zoom.V16 for Biology; Carl Zeiss AG) and with a scanning electronic microscope (SEM EVO® 40; Carl Zeiss AG). Samples were observed hydrated for optical microscopy and dehydrated (5 h at 50 °C in oven) for scanning electronic microscopy (SEM).

### 2.9 Gel viscosity measurement

Gels shear viscosity was determined to characterize the rheological properties of prepared hydrogels before and after the growth test. The test was performed on the *Bb*/Gel samples presenting similar conidia concentrations but different CM%. The gel samples (i.e., gel samples with and without *Bb*) were harvested from the bottles containing the Bb/Gel samples and the shear viscosity was measured with a single shear rate test with a Malvern Kinexus Pro rotational rheometer in a plate–plate configuration (50 s^−1^ shear rate, temperature 30 °C, atmospheric pressure). Shear viscosity was evaluated since it is related to the structural integrity of the polymer, which could be altered by the aging of the polymer or by the action of the fungus, affecting relevant properties for the proper functioning of the substrate (e.g., water retention, yield stress, texture, etc.).

### 2.10 Statistical analysis

Ordinary one‐way and two‐way ANOVA analyses were performed using Graphpad Prism 8 software. The analyses of variance were followed by Tukey's *post hoc* multiple comparison test. The parameters used in the ANOVA analysis were *P* < 0.05 at 95% level of confidence.

## 3 RESULTS

### 3.1 *Bb* growth evaluation in the *Bb*/Gel system

**Figure 1:**
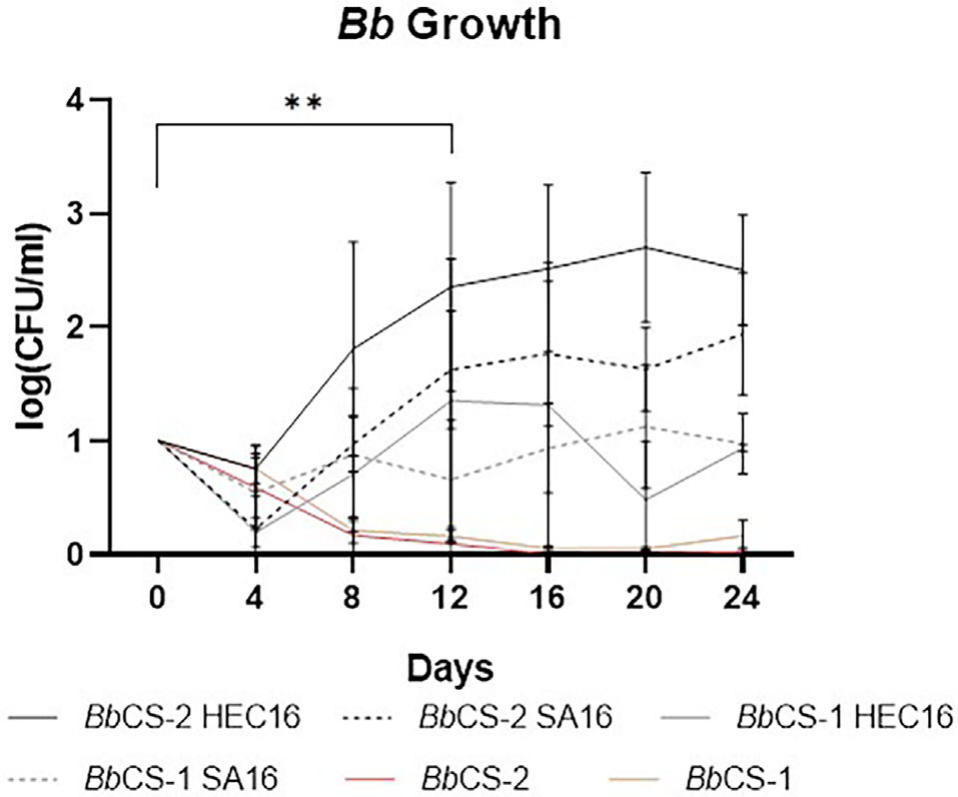
Bb growth in HEC‐ and SA‐based Bb/Gel systems prepared from CS‐1 (without nutrients) and CS‐2 (with pepton and 2% wt of chitosan as nutrients) and liquid conidial suspensions BbCS‐1 and BbCS‐2 as controls. Results expressed as log of normalized values (with respect to day 0 values) ± standard deviation, SD. Statistical analysis legend: *P* < 0.0021 = **.

**Figure 2:**
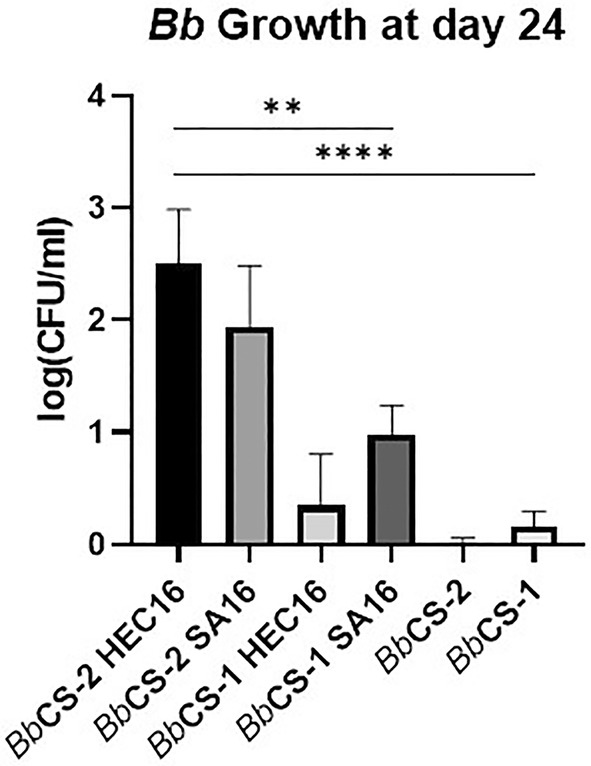
Conidial concentration comparison at day 24 for the *Bb*/Gel system (normalized CFU/mL samples 24 days ± standard deviation, SD). Statistical analysis legend: *P* < 0.0021 = **, *P* < 0.0002 = ***.

The survival and growth dynamics of *Bb* within the *Bb*/Gels are illustrated in Fig. 1. All samples exhibited an initial decrease in conidial concentration until day 4. Subsequently, the *Bb* conidial concentration in liquid suspension (i.e., *Bb*CS‐1 and *Bb*CS‐2) decreased to near zero within 16 days (−99% for CS‐2) and increased within all gel systems. Among the gel formulations, *Bb*CS‐2 gels (i.e., *Bb*CS‐2 HEC16, *Bb*CS‐2 SA16) exhibited consistently higher CFU/mL values than those derived from *Bb*CS‐1 (Fig. 2). Specifically, after 24 days, the conidial concentration in *Bb*CS‐2‐based systems was approximately one order of magnitude higher than in the *Bb*CS‐1‐based systems. *Bb*CS‐2 HEC16 recorded higher CFU/mL values at each measured time point compared to *Bb*CS‐2 SA, but this difference lacked statistical significance. The growth rate of *Bb* was significantly higher in *Bb*CS‐2 HEC16 compared to the other gels systems and controls. Remarkably, from day 8 onwards, *Bb*CS‐2 HEC16 exhibited a conidial concentration significantly exceeding that of the control groups. No growth of *Bb* was found in CS‐1 and CS‐2 (i.e., controls without *Bb*).

**Figure 3:**
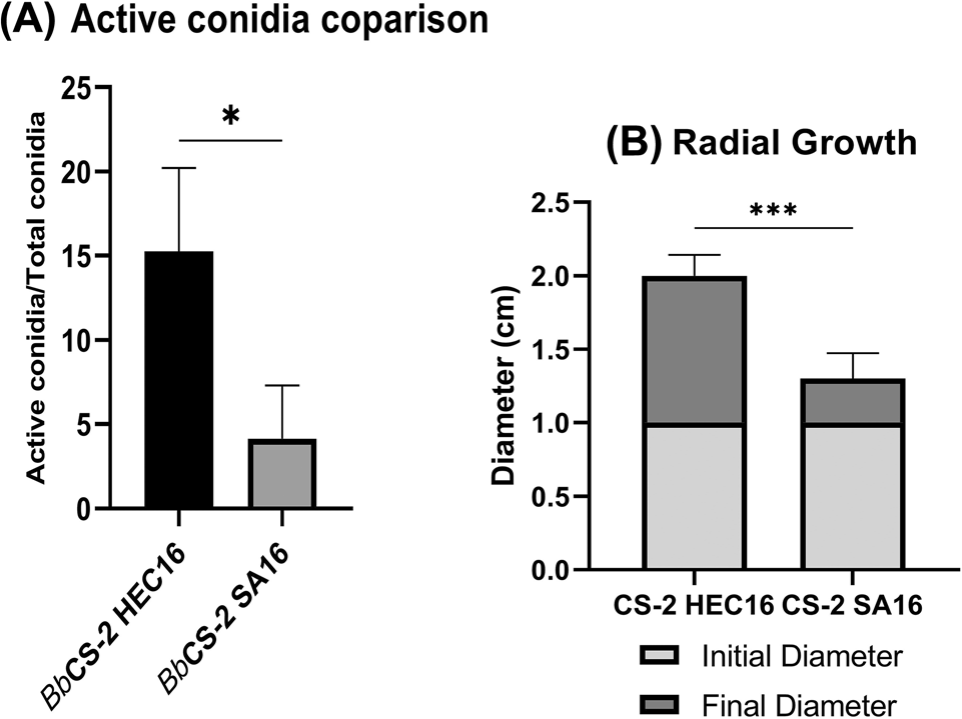
(A) Active conidia % ± Standard Deviation (SD) of *Bb*/Gel systems BbCS‐2 HEC16 and BbCS‐2 SA16 (24 days old). (B) Final mycelium colony diameters (cm) obtained by putting *Bb* punches (Initial diameter = 1 cm) for 10 days at 25 °C, 90% RH on CS‐2 HEC16 and CS‐2 SA16 as growth substrates. Statistical analysis legend: *P* < 0.033 = *; *P* = 0.0002 = ***.

*Bb*/Gel CS‐2 HEC16 had active conidia values three times higher than those of the other gels tested (Fig. 3(A)). A consistently higher mycelium development was observed when the 10‐mm diameter mycelium plug was placed in contact with CS‐2 HEC16 compared to the other gels tested (Fig. 3(B)).

### 3.2 *Bb*/gel effect on *Ae. albopictus* egg hatching

**Figure 4:**
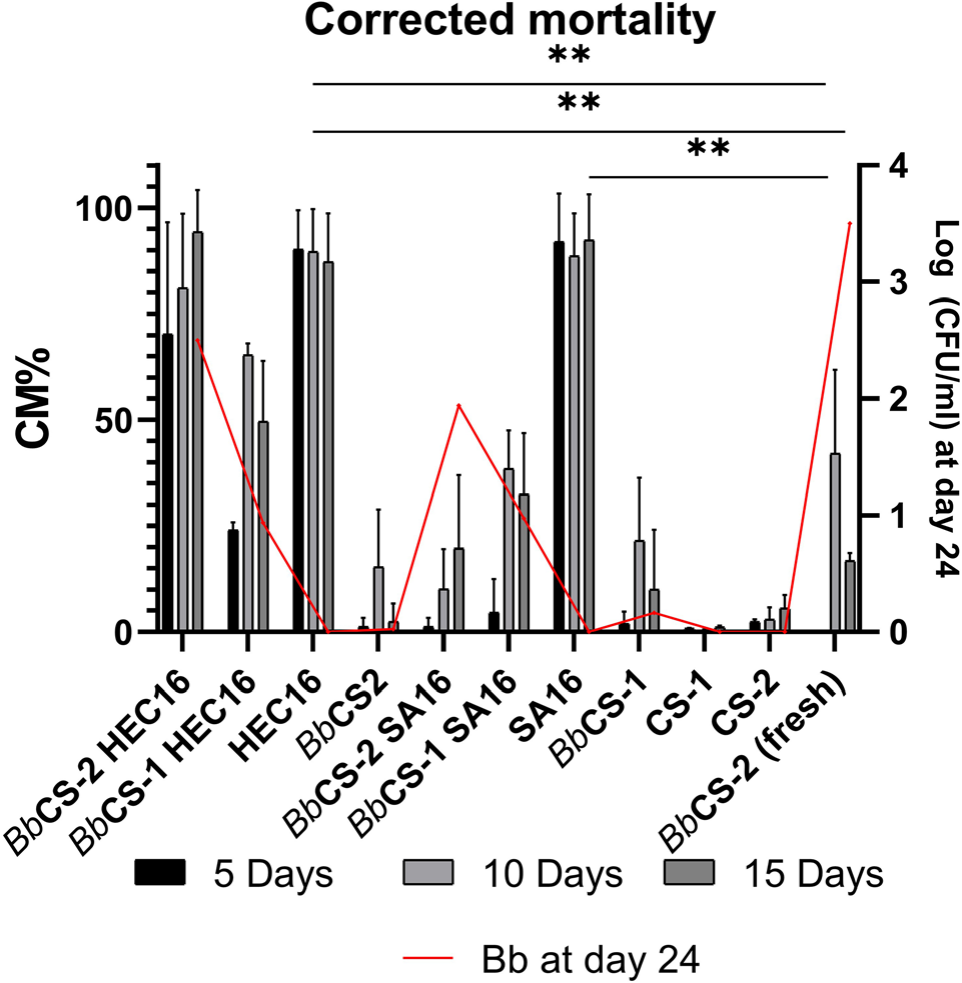
Corrected mortality (CM%) at different contact times (5, 10 and 15 days) in 24‐day‐old *Bb*/Gel systems, gel controls (HEC16, SA16), liquid controls (CS‐1, CS‐2, *Bb*CS‐1, *Bb*CS‐2), and fresh *Bb*CS‐2. CM% was calculated using distilled water as reference. CFU/mL of conidia on day 24 of growth test is reported on the right axis to evaluate conidial concentration effect on CM%. Results expressed as CM% ± SD. Statistical analysis legend: *P* < 0.0021 = **.

The virulence of the *Bb*/Gel systems on *Ae. albopictus* eggs post fungal growth is reported in Fig. 4. In particular, HEC‐based *Bb*/Gel systems are more effective than those based on SA, regardless of whether they are prepared with *Bb*CS‐2 or *Bb*CS‐1. Contact period impact mortality and the highest CM% were registered at 10 days post infection in *Bb*/Gel systems. A statistically higher CM% of *Ae. albopictus* eggs was registered by using *Bb*CS‐2 HEC16 (CM% at 15 days = 94%) than those registered in the other gel systems and controls. A higher mortality of *Ae. albopictus* eggs was also registered in gel controls (gels without *Bb* i.e., HEC16, SA16). Fresh *Bb* conidial suspension (24 h‐old *Bb*CS‐2) was more effective than *Bb*CS‐1 and *Bb*CS‐2 but significantly less active than HEC16, SA16, and *Bb*CS‐2 HEC16.

### 3.3 Morphological assays on *Bb*/Gel systems

**Figure 5:**
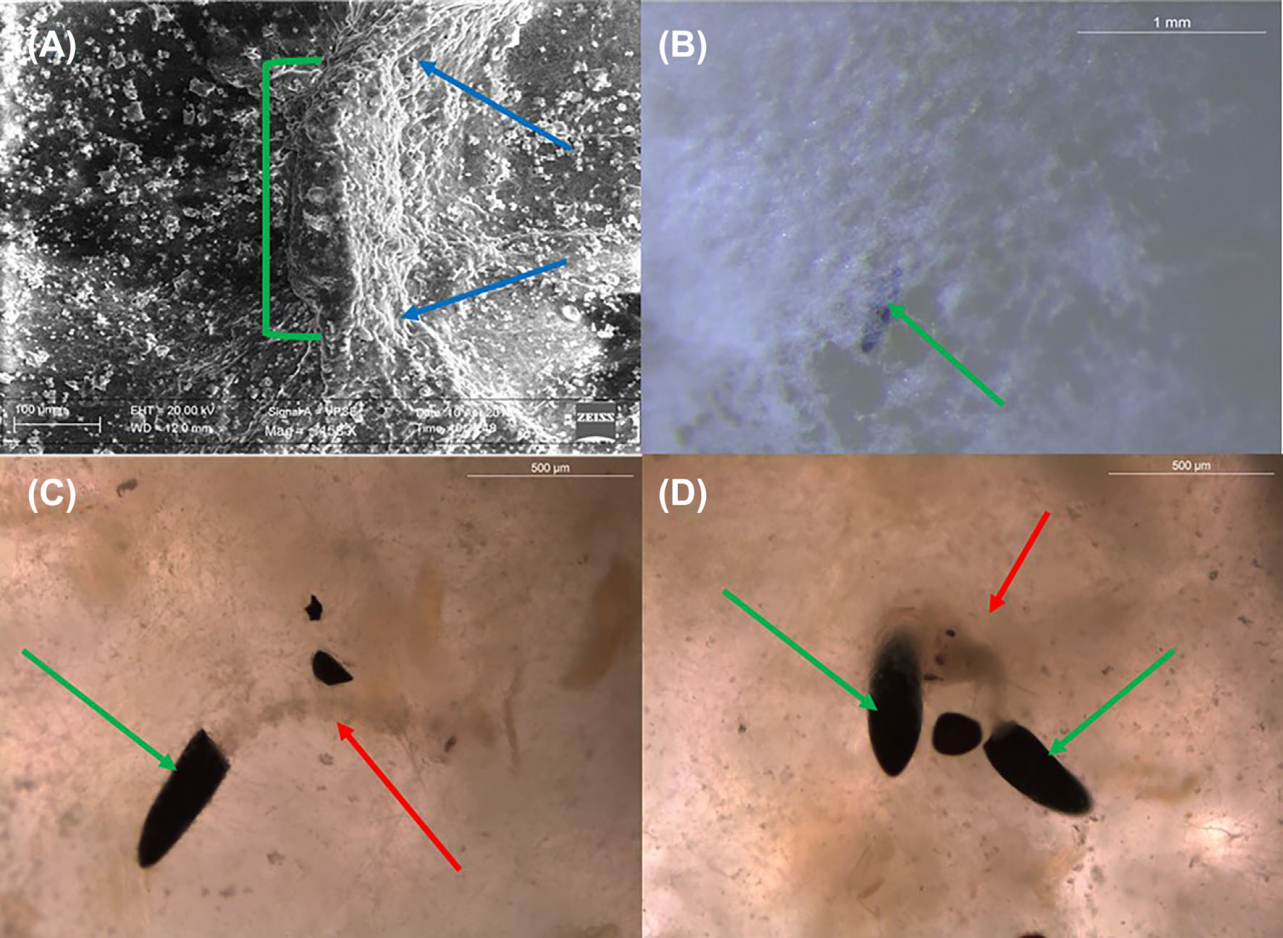
Morphological analysis of sample CS‐2 HEC16 (5 days contact time). (A) Scanning electronic microscopy images (scale bar 100 μm) of egg (green bracket) covered by fungal system (blue arrows). (B) Optical microscope observations: mycelium on egg (green arrow, scale bar 1 mm). (C, D) Optical microscope analysis (scale bar 500 μm): dead larvae trapped (red arrows) inside the gel and eggs (green arrows).

SEM and optical microscope observations were performed on eggs to evaluate the effect of HEC16, conidia, and mycelium on CM%. Morphological assays (Fig. 5) showed the contemporary action of different mechanisms: HEC16 mechanical action of entrapment on first‐instar larvae and egg infection by *Bb*. The SEM images (Fig. 5(A)) show the emergence of fungal structures (blue arrows) from an egg (green bracket) after 5 days of contact in *Bb*CS‐2 HEC16 and the presence of the mycelium that traps eggs and eventually larvae (Fig. 5(B), green arrow). Finally, Fig. 5(C),(D) shows trapped larvae (red arrows) just after hatching from an egg, which were unable to move to the surface to breathe (see Supporting Information Videos).

### 3.4 Rheological assays on *Bb*/Gel systems

**Figure 6:**
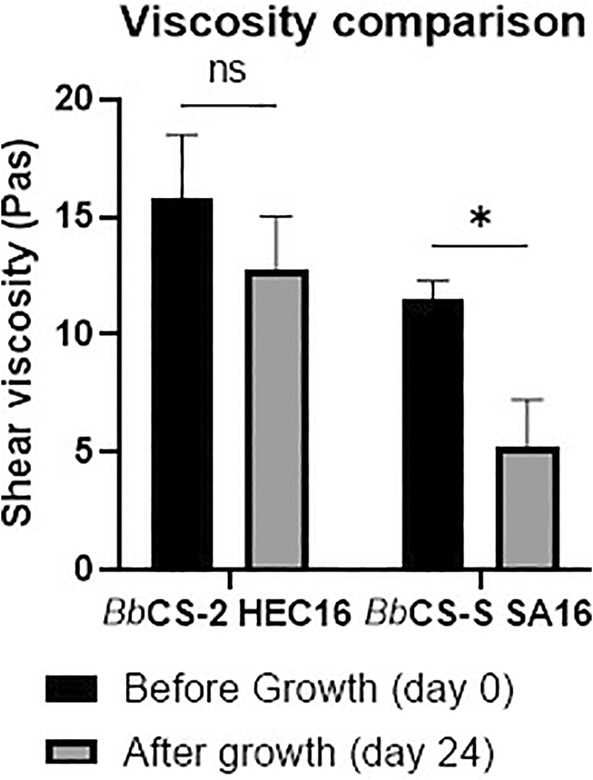
Shear viscosity ± standard deviation of *Bb*/Gel *Bb*CS‐2 HEC16 and *Bb*CS‐2 SA16 before and after they underwent growth assay (24 days at 25 ± 1 °C and 90% RH). Statistical analysis legend: *P* < 0.0123 = not significant (ns), *P* < 0.033 = *.

The shear viscosity of *Bb*/Gel systems (i.e., *Bb*CS‐2 HEC 16 and *Bb*CS‐2 SA16) was evaluated at the beginning (day 0) and at the end of the growth (day 24). A reduction in viscosity values between the two time points was found and a higher reduction in viscosity was registered in *Bb*CS‐2 SA16SA samples than in *Bb*CS‐2 HEC 16 samples (−72% and −25%, respectively; Fig. 6).

## 4 DISCUSSION AND CONCLUSION

The results of this study show that the tested hydrogel formulations can be successfully employed as growth matrix for *Bb* and, contemporarily, as a lethal substrate against *Ae. albopictus* eggs and larvae. Gel systems enhance *Bb*'s longevity (up to 24 days) more effectively than liquid conidial suspensions, suggesting that the tested HEC‐ and SA‐based hydrogels are not toxic for the fungus (biocompatibility) and create a microenvironment suitable to sustain prolonged growth of *Bb*, most likely due to their ability to prevent desiccation and maintain moisture for extended periods.^43^ However, it can also be assumed that fungal structures can interact more effectively with a solid‐like substrate compared to water. Such a phenomenon has already been observed in the medical field.^50^ For example, scaffolds for cell regeneration must possess mechanical properties comparable to those of the tissue from which the cells originate, otherwise vitality and proliferation are inhibited.^46^, ^51^ The positive effect of the gel is further enhanced when it is made by using a conidia solution containing nutrients such as peptone and chitosan that can be a valid supplementary source of chitin.^52^ In fact, CS‐2‐based *Bb*/Gel systems outperform CS‐1‐based *Bb*/Gel systems by not only sustaining vitality but also fostering growth. Polymer type influences the growth dynamics of *Bb*. Higher CFU/mL values in *Bb*CS‐2 HEC16 than *Bb*CS‐2 SA16 might be due to the higher viscosity of HEC compared to SA, which also affects *Bb* vegetative growth and conidia viability.^53^

In theory, the finding that *Bb*CS‐2 HEC16 was the most effective in promoting a lethal effect in *Ae. albopictus* eggs might be due to the higher *Bb* conidial concentration at the beginning of hatching test (*t*_0_). However, since high mortality was also observed in gel controls without *Bb* (i.e., HEC16 and SA16), the *Ae. albopictus* egg mortality cannot be solely attributed to the fungal action but also to other lethal mechanisms strictly linked to the hydrogel and acting in combination with the fungus.

The lethal effect of both *Bb*/Gel systems and gel controls HEC16 and SA16 might be attributed to a mechanical trapping effect on *Ae. albopictus* eggs and larvae due to their viscosity.^54^, ^55^ This is supported by morphological analysis on eggs and larvae in the gels and by the fact that a reduction in viscosity of the gels was registered between the beginning and the end of the growth period. In particular, the addition of *Bb* to the gel formulations reduces gel viscosity, as highlighted by rheological analysis, most likely due to the degradation of polymers by the fungus, which might have the ability to break polymer's chains (made of polysaccharides), thus lowering the polymer's molecular weight and, consequently, the viscosity.^53^, ^56^, ^57^ This phenomenon translates into a lower effectiveness of the gel with a higher reduction in viscosity and might explain the higher lethality effect on *Ae. albopictus* eggs of *Bb*CS‐2 HEC than those registered in SA‐based *Bb*/Gel systems. However, the higher mortality of *Ae. albopictus* eggs in *Bb*CS‐2 HEC after 15 days of contact, compared to that observed in gel without *Bb* (i.e., HEC16, SA16), suggests that the entrapment effect of the gel is compensated by the action of *Bb* starting from day 10. In addition, the presence of active mycelium on the eggs suggests the potential for fungal autodissemination.^33^

In conclusion, the results indicate that CS‐2 HEC16 gel is the most suitable delivery substrate for supporting the activity of *Bb* and is simultaneously effective against *Ae. albopictus* eggs through a combined mechanism of mechanical effect and fungal action. It was also observed that mechanical action alone makes the gel effective against the eggs after just 5 days, while *Bb*'s action requires a longer contact period.

However, the mechanical action affects only the insects that directly interact with the gel and the inclusion of *Bb* allows adult females in traps to be targeted and, at the same time, could lead to the autodissemination of the entomopathogen within oviposition sites, creating a trap that can be also a contamination station, enhancing the approach's effectiveness.^58^, ^59^ The impact of the *Bb*/Gel system on adult mosquitoes needs to be investigated in terms of attraction, oviposition, mortality, and potential autodissemination. Another aspect to consider is the potential contamination of the substrate by other fungi or bacteria when it is used in the field, and how this could influence (either positively or negatively) the activity of *Bb* as well as the attractiveness of the substrate.

Finally, this study provides a proof of concept for the use of a biocompatible biomimetic hydrogel in combination with various natural biocides and target pests. By adjusting the material's properties and composition, the hydrogel is established as a versatile platform for precision pest management.

## Supporting information


**Supporting Information Videos**.

## Data Availability

The data that support the findings of this study are available from the corresponding author upon reasonable request.
